# Recompensation of Liver Cirrhosis by TIPS Reduces Epithelial Cell Death Markers, Translating Into Improved Clinical Outcome

**DOI:** 10.1111/liv.16156

**Published:** 2024-11-12

**Authors:** Felix Piecha, Beatrice‐Victoria Jahn, Johannes Köntopf, Anja Koop, Ann‐Kathrin Ozga, Amirah Al‐Jawazneh, Aenne Harberts, Christoph Riedel, Peter Buggisch, Daniel Benten, Peter Hübener, Gerhard Adam, Samuel Huber, Ansgar W. Lohse, Peter Bannas, Johannes Kluwe

**Affiliations:** ^1^ Department of Medicine University Medical Center Hamburg‐Eppendorf Hamburg Germany; ^2^ German Center for Infection Research (DZIF), Partner Site Hamburg‐Lübeck‐Borstel‐Riems Hamburg Germany; ^3^ Center for Experimental Medicine, Institute of Medical Biometry and Epidemiology University Medical Center Hamburg‐Eppendorf Hamburg Germany; ^4^ Protozoa Immunology, Bernhard Nocht Institute for Tropical Medicine Hamburg Germany; ^5^ Department of Diagnostic and Interventional Radiology and Nuclear Medicine University Medical Center Hamburg‐Eppendorf Hamburg Germany; ^6^ Ifi‐Institute for Interdisciplinary Medicine Hamburg Germany; ^7^ Department of Gastroenterology Asklepios Hospital Harburg Hamburg Germany

**Keywords:** ACLF, acute‐on chronic liver failure, DAMPs, m30, m65, portal hypertension, transjugular intrahepatic portosystemic shunt

## Abstract

**Background and Aims:**

Portal hypertension is the main pathophysiological driver of decompensation in patients with liver cirrhosis. Epithelial cell death markers, m30 and m65, correlate with hepatic injury and predict outcomes across various stages of liver disease. We aim (i) to evaluate whether portal hypertension itself contributes to liver outcome‐relevant epithelial injury, and (ii) to analyse the capacity of m30/m65 to predict outcome in patients receiving a transjugular intrahepatic portosystemic shunt (TIPS) for refractory ascites.

**Methods:**

Sixty‐six patients undergoing TIPS placement for refractory ascites and 20 patients with compensated cirrhosis as controls were prospectively enrolled in this monocentric cohort study. Epithelial cell death markers were analysed pre‐TIPS, as well as 1–3 and 6–9 months post‐TIPS. The capacity of baseline levels of m30/m65 in predicting six‐month transplant‐free survival rates was analysed by multivariable Cox proportional hazards regression.

**Results:**

Levels of m30 and m65 were higher in patients with decompensated cirrhosis (pre‐TIPS) compared with compensated cirrhosis (controls). Following correction of portal hypertension by TIPS and recompensation, both markers decreased over time, reaching levels comparable to patients with compensated cirrhosis. On multivariable analysis, pre‐TIPS baseline levels of m30 and m65 were not predictive for six‐month survival.

**Conclusion:**

Correction of portal hypertension via TIPS reduces levels of epithelial cell death markers, indicating that portal hypertension is a driver of outcome‐relevant, hepatic cell death in patients with decompensated cirrhosis. Baseline m30/m65 values do not affect six‐month survival rates, which suggests that TIPS placement overcomes the unfavourable spontaneous prognosis otherwise indicated by elevated baseline m30/65 levels.

**Summary**

- Epithelial cell death markers m30 and m65 are elevated in patients with decompensated liver cirrhosis and predict liver‐relevant outcome.
- In this study, we show that correction of portal hypertension by a transjugular intrahepatic portosystemic shunt (TIPS) reduces levels of epithelial cell death markers and overcomes the unfavourable spontaneous prognosis otherwise indicated by elevated baseline m30/m65 levels.

**Abbreviations**
ACLF: acute‐on‐chronic liver failure
AD: acute decompensation
ALD: alcohol‐related liver disease
ASH: alcoholic steatohepatitis
cK18: caspase‐cleaved keratin 18
DAMP: damage‐associated molecular pattern
ePTFE: expanded polytetrafluoroethylene
FIPS: Freiburg index of post‐TIPS survival
FU: follow‐up
HBV: hepatitis B virus
HCC: hepatocellular carcinoma
HMGB‐1: high‐mobility‐group‐box 1
HRS: hepatorenal syndrome
K18: keratin 18
MELD: model for end‐stage liver disease
PSPG: porto‐systemic pressure gradient
TIPS: transjugular intrahepatic portosystemic shunt

## 1 Introduction

Advanced chronic liver disease is characterised by the transition from an asymptomatic to a symptomatic decompensated state. Portal hypertension has been identified as a key driver of decompensating events in patients with compensated liver cirrhosis [1]. Decompensation of liver cirrhosis is associated with an increased risk of further decompensation [2], development of acute‐on‐chronic liver failure (ACLF) [3] and, ultimately, increased mortality [4]. Ongoing liver injury due to an untreated underlying chronic liver disease induces further hepatic scarring, and thus, promotes portal hypertension as the crucial pathomechanism of clinical hepatic deterioration [5]. Moreover, it has also been established that the continuous release of damage‐associated molecular patterns (DAMPs) from dying hepatocytes promotes progression of portal hypertension via activation of the innate immune system. This contributes to systemic inflammation characteristic of end‐stage liver disease, and thus, leads to a self‐perpetuation of decompensation [6, 7].

Keratin 18 (K18) and caspase‐cleaved K18 (cK18) are established markers of epithelial cell death [8], with a well‐documented association with liver damage and fibrosis stages in various etiologies [9, 10, 11, 12, 13, 14] and clinical outcomes in liver cirrhosis [15, 16, 17, 18, 19, 20]. Keratins are the main epithelial subgroup of intermediate filament proteins, and K18 is—together with K8—the main intermediate filament in liver cells [21, 22, 23]. Early in apoptosis, caspases are activated to cleave K18, generating a neoepitope that is recognised by the m30 antibody, which is generally considered to reflect hepatic apoptosis [8, 24]. Both intact and fragmented K18 variants are recognised by the m65 antibody so that m65 is considered to reflect necrotic or rather overall cell death [25].

DAMP levels have been associated with portal hypertensive decompensation in liver cirrhosis by observational data [20]. However, it is unclear whether the relation between liver injury reflected by DAMPs and progression of portal hypertension is solely unidirectional. Given the correlation between DAMPs and complications of portal hypertension, our aim was to study the hypothesis that portal hypertension itself injures the liver, and thus, self‐perpetuates disease progression, which ultimately leads to hepatic decompensation and death. If this was true, relieving portal pressure should reduce markers of ongoing liver injury and improve the clinical outcome in patients with decompensated liver cirrhosis.

The implantation of a transjugular intrahepatic portosystemic shunt (TIPS) for refractory ascites represents an ideal model to study the pathophysiological changes associated with portal hypertension, as patients are (i) in a stable decompensated state with a controlled etiological factor, and (ii) receive a TIPS as an elective procedure in the absence of an acute liver‐related event or bacterial infection.

In this prospective study, we therefore evaluated whether correction of portal hypertension in patients with liver cirrhosis attenuates liver outcome‐relevant cellular injury. For this purpose, we analysed epithelial cell death markers before and after TIPS implantation and studied their association with clinical outcome in patients with decompensated portal hypertension.

## 2 Materials and Methods

### 2.1 Patients

This monocentric, prospective observational and explorative study was approved by the local ethics committee (reference number PV5727) in accordance with the principles of the declaration of Helsinki. All patients with liver cirrhosis who received an elective TIPS at the University Medical Center Hamburg‐Eppendorf for recurrent or refractory ascites between 09/2018 and 12/2020 were screened, and written informed consent was obtained prior to inclusion. All patients underwent a standardised pre‐TIPS evaluation including abdominal imaging (ultrasound and/or CT scan), echocardiography, laboratory work‐up and a standardised interview for a detailed disease history, including alcohol consumption, the time point of the first ascitic decompensation and monthly paracentesis frequency.

After TIPS placement, all patients underwent a standardised follow‐up protocol that included outpatient visits 4–12 weeks (early follow‐up [FU]) and 3–6 months (late FU) after the procedure, and then at least every 6 months, depending on clinical necessity. During these visits, abdominal ultrasound and blood analysis were performed. If patients missed their scheduled follow‐up visit, patients and their primary care physician were contacted by phone.

To assess the clinical significance of systemic cell death markers measured before and after TIPS implantation, relevant clinical outcomes were predefined as, (i), therapeutic success of TIPS defined as ascites control, and (ii), transplant‐free survival at 6 months. Accordingly, patients were characterised as TIPS nonresponders if they had persistent ascites post‐TIPS, defined as severe ascites requiring paracentesis after more than 3 months after TIPS placement, as described before [26, 27]. Patients were also classified as TIPS nonresponders if they died or underwent liver transplantation sooner than 3 months after TIPS placement while still exhibiting ascites requiring paracentesis. Patients who showed a secondary response after a TIPS revision were included in the ascites control group for the clinical analysis, even if the need for paracentesis exceeded the 3 months threshold. However, for the longitudinal evaluation of epithelial cell death markers before and after TIPS, patients who still required paracentesis at the time point of blood sampling during the early FU visit (4–12 weeks post‐TIPS) were considered nonresponders at that specific time and for this specific analysis only, even if they showed a secondary response after a reintervention.

Patients with stable compensated liver cirrhosis as controls were recruited from the outpatient clinic of the University Medical Center Hamburg‐Eppendorf and were also followed up every 6 months.

### 2.2 TIPS Procedure

All TIPS procedures were performed by experienced interventional radiologists. Sedation was carried out with midazolame (Hofmann‐La Roche, Basel, Switzerland) and piritramide (Hameln pharma plus GmbH, Hameln, Germany) as described before [26]. All patients underwent invasive pressure measurement in the superior vena cava and portal vein with calculation of the subsequent porto‐systemic pressure gradient (PSPG). A 10 mm expanded polytetrafluoroethylene (ePTFE) coated stentgraft (VIATORR, W.L. Gore & Associates Inc., AZ, USA) was dilated to 8 mm using an 8/40 mm balloon dilatation catheter (Boston Scientific, Marlborough, MA, USA) in all cases.

### 2.3 Study Blood Samples

Blood samples that were utilised for the study were drawn together with blood for routine laboratory analyses from the cubital vein in the morning of the TIPS procedure, as well as before discharge and during the FU visits in the outpatient clinic, as mentioned above. Study blood was also obtained from control patients with compensated cirrhosis. Samples were centrifuged at 400 *g* for 5 min the same day, and serum and plasma were stored at −80° Celsius until further analysis.

### 2.4 Measurement of Epithelial Cell Death Markers and High‐Mobility‐Group‐Box 1 (HMGB1)

For the quantitative measurement of epithelial cell death markers, the m30‐Apoptosense and m65 EpiDeath ELISA (both PEVIVA, Teco Medical group, Sissach, Switzerland) were used according to the manufacturer's guidelines. The m30:m65 ratio (apoptotic index) was calculated during statistical analysis.

Plasma levels of HMBG1 were quantified using a commercially available HMGB1 ELISA Kit (Tecan group, Männedorf, Switzerland). All samples were run in duplicates. In order to minimise batch effects, all samples from an individual patient were measured on a single ELISA plate.

### 2.5 Statistical Analysis

Percentages and counts are given for categorical data, median values with the corresponding 0.25‐ and 0.75 quartile for continuous variables. Due to the small sample size and skewed data, two or more independent continuous variables were compared using a Mann–Whitney‐*U*‐ or Kruskal–Wallis Test and two or more dependent continuous variables were compared with a Wilcoxon‐ or Friedman‐Test. Correlation analysis was carried out using Spearman's Rho. In addition, a multivariable Cox proportional hazards model to identify predictors of six‐month transplant‐free survival was carried out including variables that were considered outcome‐relevant by the authors. For this analysis, age, sex and baseline values of the model for end‐stage liver disease (MELD) score (log scale), Freiburg index of post‐TIPS survival (FIPS), m30 (log scale) and m65 (log scale) were used. Given the exploratory character of the analysis, no sample‐size calculation was carried out and no adjustment for multiple testing was conducted. *p* values are also considered explorative. Missing values were not imputed. Statistical testing was carried out using SPSS Version 25 (IBM, Armonk, NY, USA), R Version 4.1.2 (R Core Team, 2021) and GraphPad Prism Version 9.4.1 (Graphpad Software, San Diego, CA, USA).

## 3 Results

### 3.1 Cohort Overview

A total of 66 patients undergoing scheduled TIPS placements for recurrent or refractory ascites were included in this study. Samples acquired during early (1–3 months) and late (3–6 months) follow‐up visits in our outpatient clinic post‐TIPS were available in 40/66 (61%) and 32/66 patients (48%), respectively. Information on the predefined relevant clinical outcomes were available in (i) 61/66 patients (92%) for ascites control and (ii) in all patients regarding overall survival. For the control group, 20 patients with compensated liver cirrhosis were recruited and prospectively followed up by our outpatient clinic.

### 3.2 Baseline Characteristics and Outcome Parameters

In the TIPS cohort, 36/66 patients (55%) were male, and the median age ranged between 58.5 (54.0, 65.0) years. Alcohol‐related liver disease (ALD) was the most common underlying aetiology, with a prevalence of 46/66 cases (70%).

TIPS was placed at a median of 29.1 (13.5, 50.1) weeks after the first ascitic decompensation. The median paracentesis frequency was 2.0 (1.0, 2.1) paracentesis per month. Accordingly, no patient presented with Child–Pugh class A at the time of TIPS placement, 51/66 patients (77%) were classified as Child–Pugh B and 15/66 patients (23%) as Child–Pugh C. The median MELD score amounted to 13.0 (10.0, 16.0) points.

Portal pressure was reduced from a median of 25.0 (21.0, 28.0) to 10.0 (7.0, 13.0) mmHg by TIPS. Median pressure reduction amounted to 15.0 (11.0, 18.5) mmHg or − 60 (−71, −47) percent. Baseline characteristics are displayed in Table 1.

**TABLE 1:** Baseline characteristics and outcome parameters in 66 prospectively recruited patients undergoing TIPS insertion for refractory ascites.

Baseline characteristics	Prospective TIPS cohort, *n* = 66
Sex: male/female (*n*, %)	36 (54.5)/30 (45.5)
Age [year]	58.5 (54.0, 65.0)
BMI [kg/m^2^]	26.5 (21.8, 29.4)
Aetiology (*n*, %)	
ALD	46 (69.7)
NASH	8 (12.1)
Cryptogenic	8 (12.1)
Other	4 (6.1)
Ongoing alcohol consumption (*n*, %)	9 (13.6)
Time lapse diagnosis cirrhosis—TIPS [month]	16.8 (8.1, 62.9)
Time lapse first ascites—TIPS [week]	29.1 (13.5, 50.1)
Paracentesis frequency [*n*/month]	2.0 (1.0, 2.1)
History of SBP (*n*, %)	22 (33.3)
History of HRS (*n*, %)	23 (34.8)
History of HE (*n*, %)	16 (24.2)
History of upper GI bleeding (*n*, %)	27 (40.9)
Variceal size pre‐TIPS: 0/I/II/III/IV/n/a (*n*, %)	13 (19.6)/33 (50.0)/11 (16.6)/5 (7.7)/0 (0.0)/4 (6.1)
Red spots: yes/no/unknown (*n*, %)	9 (13.6)/52 (78.7)/5 (7.7)
Child–Pugh stage A/B/C (*n*, %)	0 (0.0)/51 (77.3)/15 (22.7)
Child–Pugh score [points]	9.0 (8.0, 9.0)
MELD score [points]	13.0 (10.0, 16.0)
CLIF‐C AD score [points]	48.5 (44.0, 51.3)
FIPS score [points]	0.12 (−0.58, 0.59)
PSPG pre‐TIPS [mmHg]	25.0 (21.0, 28.0)
PSPG post‐TIPS [mmHg]	10.0 (7.0, 13.0)
ΔPSPG: absolute values [mmHg]	15.0 (11.0, 18.5)
ΔPSPG: percentage	−60.0 (−71.2, −47.0)
Outcome parameters	
Any paracentesis after TIPS: yes/no/n/a (*n*, %)	40 (60.6)/22 (33.3)/4 (6.1)
Persistent ascites: yes/no/n/a (*n*, %)	13 (19.7)/48 (72.7)/5 (7.6)
Primary response (*n/N*, %)	43/48 (89.6)
Secondary response (*n/N*, %)	5/48 (10.4)
Incidence of HE requiring hospitalisation (*n*, %)	25 (37.9)
Incidence of infections req. hospitalisation (*n*, %)	24 (36.4)
Incidence of SBP (*n*, %)	6 (9.1)
Incidence of new‐onset peripheral edema (*n*, %)	18 (27.3)
Incidence of cardiomyopathy (*n*, %)	1 (1.5)
Incidence of HCC (*n*, %)	1 (1.5)
Incidence of liver transplantations (*n*, %)	3 (4.5)
Incidence of death or LT at 6 months (*n*, %)	19 (28.8)
Overall incidence of death or LT (*n*, %)	28 (42.4)
Transplant‐free survival [mo]	14.9 (5.2, 23.3)

*Note:* Data are shown as counts and percentages as indicated or median values with the 0.25‐ and 0.75‐quartile.

Abbreviations: ALD, alcohol‐related liver disease; BMI, body mass index; CLIF‐C AD, chronic liver failure consortium acute decompensation; FIPS, Freiburg Index of post‐TIPS survival; GI, gastrointestinal; HCC, hepatocellular carcinoma; HE, hepatic encephalopathy; HRS, hepatorenal syndrome; LT, liver transplantation; MELD, model for end‐stage liver disease; NASH, non‐alcoholic steatohepatitis; PSPG, portosystemic pressure gradient; SBP, spontaneous bacterial peritonitis; TIPS, transjugular intrahepatic portosystemic shunt.

After TIPS placement, 40/61 patients (66%) required any paracentesis. Ascites control was achieved in 48/61 patients (79%), of which 43 patients (90%) showed a primary response, and 5/48 patients (10%) a secondary response either after TIPS dilatation (*n* = 4) or spontaneously (*n* = 1). TIPS refractory ascites, defining TIPS non‐response, was diagnosed according to the abovementioned criteria in 13/61 patients (21%). In the remaining five patients, ascites control could not be classified due to unclear endpoint interpretation in one patient because of alternating phases of recompensation and decompensation, presumably caused by ongoing alcohol consumption, and missing information on ascites control in the four other patients due to incomplete data acquisition during follow‐up. These five patients were excluded from the analysis regarding persistent ascites post‐TIPS.

During follow‐up, 3/66 patients (5%) underwent liver transplantation, and 25/66 patients (38%) died, resulting in a median transplant‐free survival of 14.9 (5.2, 23.3) months. The incidence of liver transplantation (*n* = 3) or death (*n* = 16) at 6 months was 19/66 patients (29%). Outcome parameters are also detailed in Table 1.

For the control cohort, 20 patients with compensated liver cirrhosis were recruited from our outpatient clinic. Similar to the TIPS cohort, patients were predominantly male (15/20 patients, 75%) and had a median age of 63.0 (54.2, 70.6) years. The most common underlying aetiology was also ALD, which was observed in 13/20 patients (65%). At the time of blood sampling, 18/20 patients (90%) were classified as Child–Pugh A, and 2/20 patients (10%) as Child–Pugh B. The median MELD score was 8.0 (6.3, 10.8) points, thus reflecting the clinically compensated status of liver disease. During follow‐up, no patient developed ascites, was hospitalised for hepatic encephalopathy, had an infection, or underwent liver transplantation. *Hepatocellular carcinoma* (HCC) was detected in 1/20 patient (5%), and 2/20 patients (10%) died due to liver‐unrelated reasons. Details on baseline characteristics and outcome parameters of the control cohort are given in Table S1.

### 3.3 M30 and m65 Levels Are Increased in Patients With Decompensated Liver Cirrhosis

**FIGURE 1:**
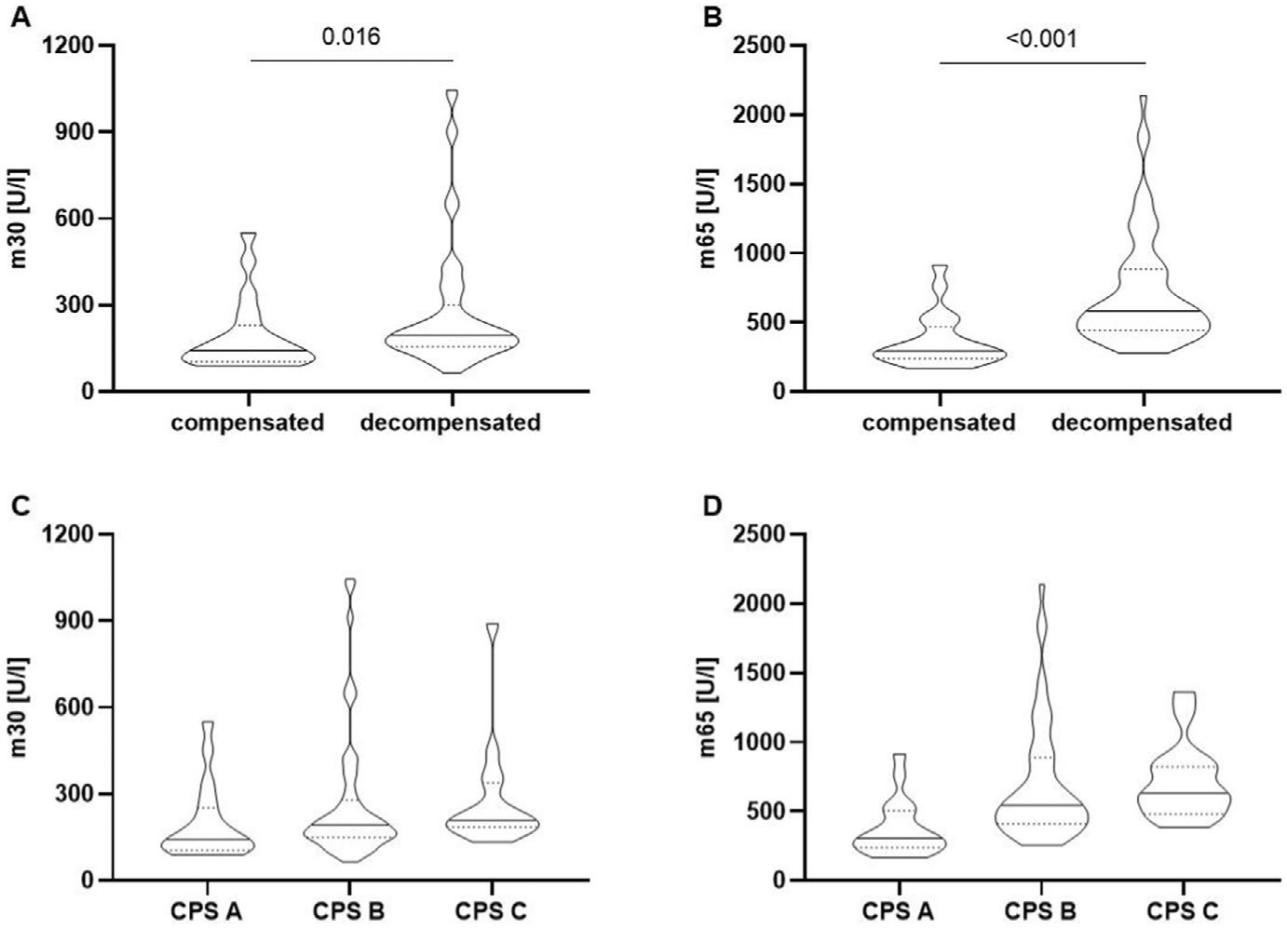
Epithelial cell death markers m30/m65 in patients with compensated and decompensated cirrhosis pre‐TIPS (A+B) and stratified according to the Child–Pugh stage (C+D). Both m30 (A) and m65 (B) levels were higher in patients with decompensated (*n* = 66) compared to compensated (*n* = 20) liver cirrhosis (*p* = 0.016 and < 0.001, respectively, Mann–Whitney‐*U* test), and showed a stepwise increase when stratified according to the Child–Pugh stage (panel C, D; CPS A: *n* = 18, CPS B: *n* = 53, CPS C: *n* = 15). Data are shown as violin plots depicting the median (solid line) and the 0.25‐ and 0.75 quartile (dotted lines). CPS, Child–Pugh score; TIPS, transjugular intrahepatic portosystemic shunt.

We first evaluated levels of epithelial cell death markers depending on the compensation status of patients. Patients with ascitic decompensated liver disease pre‐TIPS showed increased levels of both m30 and m65 compared to patients with compensated liver disease (median m30: 195.2 (156.2, 300.6) U/L vs. median 142.5 (103.5, 230.0) U/L, *p* = 0.016; median m65: 582.4 (444.3, 885.0) U/L vs. 291.1 (239.9, 468.8) U/L, *p* < 0.001 in *n* = 66 decompensated patients vs. *n* = 20 patients with compensated liver cirrhosis, Figure 1A,B, Table 2). This difference in compensated vs. decompensated liver cirrhosis was also seen for the m30:m65 ratio (apoptotic index, Table 2), suggesting a predominance of non‐apoptotic cell death in patients with decompensated liver cirrhosis.

**TABLE 2:** Levels of epithelial cell death markers in patients with compensated (*n* = 20) and decompensated liver cirrhosis (pre‐TIPS, *n* = 66).

Parameter	Compensated, control group (*n* = 20)	Decompensated, pre‐TIPS (*n* = 66)
m30 [U/L]	142.5 (103.5, 230.0)	195.2 (156.2, 300.6)
m65 [U/L]	291.1 (239.9, 468.8)	582.4 (444.3, 885.0)
m30:m65 (apoptotic index)	0.47 (0.39, 0.58)	0.36 (0.26, 0.45)

*Note:* Both individual m30 and m65 values as well as their ratio (m30:m65, apoptotic index) were relevantly different in patients with decompensated liver cirrhosis pre‐TIPS compared to patients with compensated liver cirrhosis as controls. Data are shown as median values with the 0.25‐ and 0.75‐quartile.

Abbreviation: TIPS, transjugular intrahepatic portosystemic shunt.

When stratified according to the Child–Pugh score, both m30 and m65 values showed a stepwise increase across disease stages, (m30: median increase from 142.5 (104.4, 252.1) U/L in Child–Pugh A to 191.5 (149.8, 278.3) U/L in Child–Pugh B and 209.3 (184.4, 338.1) U/L in Child–Pugh C; m65: median increase from 304.5 (237.2, 502.8) U/L in Child–Pugh A to 543.1 (411.0, 886.7) U/L in Child–Pugh B to 629.8 (479.1, 822.0) U/L in Child–Pugh C, Figure 1C,D).

Lastly, we conducted correlation analyses of baseline m30 and m65 values with other baseline laboratory values, the corresponding liver disease severity scores, and invasively measured pressure parameters. M30 and m65 values correlated with each other (Spearman's rho 0.678), but not with transaminases, liver function parameters like albumin, or pressure values (Table S2).

### 3.4 Decrease of Epithelial Cell Death Markers After TIPS Implantation

**FIGURE 2:**
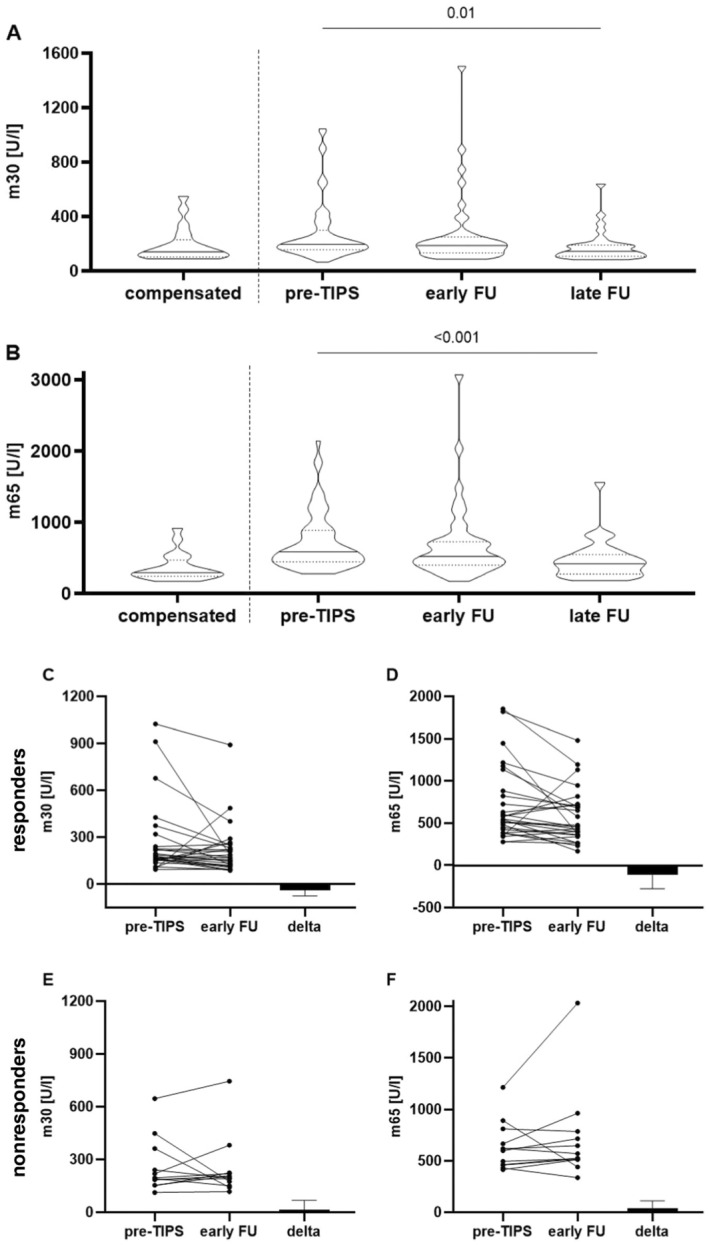
Evolvement of epithelial cell death markers m30 (A) and m65 (B) during early and late follow‐up a median 35 and 190 days after TIPS placement and stratified according to patients with ascites control (C, D) and persistent ascites (E, F) at early follow‐up. Both m30 and m65 values showed a relevant decrease over time, returning to values comparable to patients with compensated liver cirrhosis (A, B, *p* = 0.01 for m30 and < 0.001 for m65, Friedman‐Test). When stratified into patients with a response and nonresponse defined as having ascites requiring paracentesis at early follow‐up, levels of m30 and m65 only decreased in patients with a response (C, D) but remained similar in patients with a nonresponse (E, F). For the separate analysis of responders and nonresponders, patients that showed a secondary response after TIPS dilatation were also classified as nonresponders for presenting with ascites requiring paracentesis at the time of blood sampling. Number of patients at each assessment: Pre‐TIPS *n* = 66, early follow‐up *n* = 40, late follow‐up *n* = 32. Responders: *n* = 28; nonresponders *n* = 12. Panel A and B depict violin plots with the median (solid line) and the 0.25‐ and 0.75‐quartile (dotted lines).

Next, we analysed the dynamics of epithelial cell death markers after TIPS by comparing pre‐TIPS values to values determined during the first visit in our outpatient clinic at a median of 35 (27, 45) days post‐TIPS for the early follow‐up, and at a median of 190 (147, 259) days post‐TIPS for the late follow‐up. Both, m30 and m65 levels decreased relevantly over time: m30 from a median of 195.2 (156.2, 300.6) U/L pre‐TIPS to 185.9 (127.0, 230.1) U/L during the early follow‐up, and 144.1 (108.0, 190.4) U/L during the late follow‐up appointment (*p* = 0.01, Figure 2A and Table 3; pairwise comparison: pre‐TIPS vs. early FU: *p* = 0.083; pre‐TIPS vs. late FU: *p* = 0.002). The m65 levels changed from a median of 582.4 (444.3, 885.0) U/L, to 517.8 (376.4, 715.0) U/L, and 415.5 (271.2, 546.4) U/L (*p* < 0.001, Figure 2B and Table 3; pairwise comparison: pre‐TIPS vs. early FU: *p* = 0.061; pre‐TIPS vs. late FU: *p* < 0.001).

**TABLE 3:** Dynamics of epithelial cell death markers and laboratory parameters during follow‐up.

Parameter	Decompensated (*n* = 66) pre‐TIPS	Early follow‐up (*n* = 40) 35 (27, 45) days post‐TIPS	Late follow‐up (*n* = 32) 190 (147, 259) days post‐TIPS
m30 [U/L]	195.2 (156.2, 300.6)	185.9 (127.0, 230.1)	144.1 (108.0, 190.4)
Δm30 [U/L]		−18.2 (−69.7, 21.9)	−48.4 (−100.7, 2.0)
Δm30% [U/L]		−12.8 (−36.7, 12.5)	−24.5 (−42.0, 1.4)
m65 [U/L]	582.4 (444.3, 885.0)	517.8 (376.4, 715.0)	415.5 (271.2, 546.4)
Δm65 [U/L]		−59.8 (−187.7, 61.1)	−159.3 (−393.4, −48.8)
Δm65% [U/L]		−10.0 (−24.5, 13.8)	−30.0 (−47.5, −10.3)
m30:m65 ratio (apoptotic index)	0.36 (0.26, 0.45)	0.35 (0.25, 0.45)	0.37 (0.33, 0.44)
Δm30:m65 ratio		0.00 (−0.06, 0.05)	0.00 (−0.06, 0.11)
Δm30:m65 ratio %		0.08 (−16.9, 13.4)	−0.39 (−14.88, 35.58)
MELD score	13.0 (10.0, 16.0)	13.0 (11.0, 16.8)	13.0 (10.0, 16.3)
ΔMELD score		1.0 (−1.0, 2.0)	0.0 (−1.0, 4.0)
ΔMELD score %		7.4 (−11.4, 19.6)	0.0 (−8.4, 36.4)
White blood cell count [1000/μL]	5.3 (3.9, 7.5)	6.0 (4.1, 7.8)	5.5 (4.0, 8.3)
Δwhite blood cell count		0.4 (−1.1, 2.0)	−0.15 (−1.73, 1.45)
Δwhite blood cell count %		10.8 (−17.1, 46.2)	−2.6 (−24.6, 32.6)
C‐reactive protein [mg/L]	12.0 (5.0, 25.5)	9.5 (5.0, 22.5)	5.0 (4.0, 8.0)
ΔC‐reactive protein		0.0 (−10.8, 5.8)	−1.0 (−14.8, 0.0)
ΔC‐reactive protein %		0.0 (−49.8, 49.0)	−20.0 (−73.4, 0.0)
GOT [U/L]	37.0 (26.8, 49.5)	40.0 (32.0, 62.0)	34.5 (28.0, 48.3)
GPT [U/L]	20.0 (16.0, 29.3)	21.0 (15.0, 37.0)	22.5 (16.0, 30.8)
gGT [U/L]	90.5 (56.0, 175.5)	78.0 (53.0, 148.0)	76.0 (32.0, 148.8)
AP [U/L]	125.0 (87.5, 158.5)	154.0 (110.0, 222.0)	126.5 (92.8, 185.3)
Bilirubin [mg/dL]	1.4 (0.7, 2.1)	1.8 (1.2, 2.9)	1.6 (1.1, 2.7)
INR	1.3 (1.2, 1.4)	1.3 (1.2, 1.5)	1.3 (1.1, 1.5)
Creatinine [mg/dL]	1.1 (0.8, 1.6)	1.0 (0.8, 1.3)	1.1 (0.8, 1.4)
Albumin [g/L]	26.7 (23.3, 30.2)	25.3 (21.7, 29.5)	28.7 (22.0, 33.8)
Platelets [1000/μL]	99.5 (80.5, 147.5)	119.5 (86.0, 163.5)	112.0 (78.0, 186.8)
Fib‐4 score	4.46 (2.93, 6.48)	4.40 (3.00, 6.48)	4.09 (2.18, 5.41)

*Note:* During follow‐up, m30 and m65 as well as CRP levels decreased, whereas no relevant changes were seen in MELD scores and white blood cell count. Data are shown as median values with the 0.25‐ and 0.75‐quartile.

Abbreviations: CRP, C‐reactive protein; Fib‐4, fibrosis‐4; INR, international normalised ratio; MELD, model for end‐stage liver disease.

Following TIPS implantation, both markers reached levels similar to patients with compensated liver cirrhosis. Interestingly, among the standard biochemical parameters, only the C‐reactive protein (CRP) also changed relevantly from 12.0 (5.0, 25.5) mg/L at baseline to 5.0 (4.0, 8.0) at the late FU, while all other parameters remained stable (Table 3).

We then stratified the cohort at early follow‐up into two groups: TIPS responders and nonresponders. For this analysis only, a nonresponse was defined as presenting with ascites requiring paracentesis at the time of blood sampling, even if a secondary response was achieved later, either spontaneously (*n* = 1) or after TIPS revision (*n* = 4). Interestingly, a relevant change in both m30 and m65 levels was only observed in patients with a clinical TIPS response: the median decrease in m30 levels was −39.3 (−75.6, 9.2) U/L in responders (Figure 2C), while nonresponders showed a median increase of 13.8 (−45.4, 68.4) U/L (Figure 2E). For m65 levels, the median decrease in responders was −115.5 (−277.1, 30.2, Figure 2D) whereas nonresponders showed a median increase of 42.5 (−44.4, 111.1) U/L (Figure 2F).

### 3.5 Subpopulation Analysis: Stratification of the Cohort According to Post‐TIPS Ascites Control and Six‐Month Transplant‐Free Survival

To further analyse a possible prognostic influence of baseline m30 and m65 levels, we stratified the cohort according to the two predefined clinically relevant endpoints: post‐TIPS ascites control and six‐month transplant‐free survival.

Patients with persistent ascites post‐TIPS presented with a relevantly higher paracentesis frequency pre‐TIPS (2.0 (1.5, 4.0) vs. 1.7 (1.0, 2.0) paracentesis per month), a higher MELD score (17.0 (14.0, 21.5) vs. 12.0 (9.3, 14.8) points, respectively), and a higher FIPS score (0.52 (0.22, 1.16) vs. −0.15 (−0.78, 0.43) points, respectively), whereas baseline pressure levels, and the absolute and relative pressure reduction by TIPS were similar between cohorts (Table 4). Levels of m30 and m65 both showed a trend towards higher values at baseline in patients with persistent ascites post‐TIPS (Table 4 and Figure S1). Clinically, patients with persistent ascites post‐TIPS presented with a markedly increased rate of infections during follow‐up (10/13 patients, 77% vs. 13/48 patients, 28%) and incidence of death or liver transplantation at 6 months (12/13, 93% vs. 3/48, 7%), resulting in a markedly shorter transplant‐free survival (1.9 (1.1, 5.0) vs. 18.0 (12.3, 25.7) months, respectively).

**TABLE 4:** Subpopulation analysis of patients with persistent ascites vs. ascites control post‐TIPS.

Baseline characteristics	Persistent ascites, *n* = 13 (%)	Ascites control, *n* = 48 (%)
Sex: male/female (*n*, %)	11 (84.6)/2 (15.4)	21 (43.8)/27 (56.2)
Age [year]	61.0 (55.0, 65.0)	57.5 (53.3, 65.8)
Aetiology—ALD (*n*, %)	7 (53.8)	32 (66.7)
Ongoing alcohol consumption (*n*, %)	1 (7.7)	8 (16.7)
Time lapse diagnosis cirrhosis—TIPS [month]	39.3 (7.6, 95.5)	12.8 (7.6, 60.7)
Time lapse first ascites—TIPS [weeks]	25.0 (15.1, 78.8)	29.7 (10.6, 48.6)
Paracentesis frequency [*n*/month]	2.0 (1.5, 4.0)	1.7 (1.0, 2.0)
Child–Pugh stage A/B/C (*n*, %)	0 (0.0)/5 (38.5)/8 (61.5)	0 (0.0)/42 (87.5)/6 (12.5)
Child–Pugh score [points]	10.0 (9.0, 10.0)	8.5 (8.0, 9.0)
MELD score [points]	17.0 (14.0, 21.5)	12.0 (9.3, 14.8)
CLIF‐C AD score [points]	51.0 (45.5, 62.5)	48.5 (44.3, 51.0)
FIPS score [points]	0.52 (0.22, 1.16)	−0.15 (−0.78, 0.43)
PSPG pre‐TIPS [mmHg]	26.0 (23.0, 28.5)	24.5 (20.0, 28.5)
PSPG post‐TIPS [mmHg]	10.0 (7.5, 13.0)	9.0 (7.0, 12.0)
ΔPSPG: absolute values [mmHg]	15.0 (10.0, 18.0)	14.5 (11.0, 19.5)
ΔPSPG: percentage	−60.6 (−63.0, −46.4)	−60.0 (−72.0, −46.7)
Epithelial cell death markers		
Baseline m30 [U/L]	224.3 (185.8, 353.0)	182.7 (149.7, 255.6)
Baseline m65 [U/L]:	621.2 (470.2, 840.7)	557.0 (409.5, 868.0)
Baseline m30:m65 ratio (apoptotic index)	0.40 (0.26, 0.57)	0.36 (0.27, 0.44)
Outcome parameters		
Any paracentesis after TIPS: yes/no/n/a (*n*, %)	13 (100.0)/0 (0.0)/0 (0.0)	26 (54.2)/22 (45.8)/0 (0.0)
Incidence of infections req. hospitalisation (*n*, %)	10 (76.9)	13 (27.1)
Incidence of SBP (*n*, %)	2 (15.4)	3 (6.3)
Incidence of liver transplantations (*n*, %)	3 (23.1)	0 (0.0)
Incidence of death or LT at 6 months (*n*, %)	12 (92.3)	3 (6.3)
Overall incidence of death or LT (*n*, %)	12 (92.3)	12 (25.0)
Transplant‐free survival [month]	1.9 (1.1, 5.0)	18.0 (12.3, 25.7)

*Note:* Data are shown as counts and percentages as indicated or median values with the 0.25‐ and 0.75‐quartile. Patients with persistent ascites post‐TIPS presented with a higher MELD score and paracentesis frequency pre‐TIPS and overall higher values of epithelial cell death markers.

Abbreviations: ALD, alcohol‐related liver disease; CLIF‐C AD, chronic liver failure consortium acute decompensation; FIPS, Freiburg Index of post‐TIPS survival; LT, liver transplantation; MELD, model for end‐stage liver disease; PSPG, portosystemic pressure gradient; SBP, spontaneous bacterial peritonitis; TIPS, transjugular intrahepatic portosystemic shunt.

When stratified according to six‐month survival, nonsurvivors presented with higher MELD and FIPS scores at baseline compared with survivors (MELD score: 14.0 (11.0, 18.0) in nonsurvivors vs. 12.0 (9.0, 15.0) points in survivors; FIPS score: 0.42 (0.03, 0.79) in nonsurvivors vs. −0.15 (−0.81, 0.46) points in survivors, Table S3). Levels of baseline m30 and m65 also showed a trend towards higher values in nonsurvivors (Table S3 and Figure S2). With regard to outcome parameters, only one patient, who was classified with persistent ascites post‐TIPS, survived the 6 months threshold.

### 3.6 Multivariable Analysis: Baseline Values of m30 and m65 Seem Not to Predict 6‐Month Transplant‐Free Survival

Having established a trend towards higher baseline values of m30 and m65 in patients meeting the predefined relevant clinical outcomes of persistent ascites and death or LT within 6 months, we further investigated the predictive capacity of these variables. For this purpose, we incorporated baseline m30 and m65 values (both log scale) in a multivariable Cox proportional hazards model along with age, sex, baseline MELD (log scale) and FIPS scores, and for the cumulative endpoint liver transplantation and death at 6 months. In this analysis, except female sex, none of the parameters seem to be predictive for the cumulative endpoint (Table 5 and Figure S3).

**TABLE 5:** Multivariable Cox proportional hazards model to identify predictors for the cumulative endpoint death and liver transplantation at 6 months.

Predictors	Hazard ratio	95%‐confidence interval	*p*
Age	1.05	0.98–1.11	0.153
Female sex	0.25	0.08–0.84	0.025
Baseline MELD score (log scale)	1.34	0.13–13.26	0.804
Baseline FIPS score	1.93	0.55–6.84	0.307
Baseline m30 (log scale)	2.01	0.67–5.97	0.210
Baseline m65 (log scale)	0.64	0.17–2.50	0.525

*Note:* Except female sex, none of the variables that entered the model had a relevant influence on six‐month transplant‐free survival.

Abbreviations: FIPS, Freiburg Index of post‐TIPS survival; MELD, model for end‐stage liver disease.

### 3.7 Decrease in Epithelial Cell Death Markers During Early Follow‐Up Translates Into Improved Clinical Outcome

We finally stratified the cohort according to the dynamics of m30 and m65 values at early follow‐up, as baseline m30 and m65 values yielded no predictive capacity for clinical outcomes. Here, increasing values of epithelial cell death markers post‐TIPS were associated with a higher rate of ascites persistence, and impaired six‐month survival rates: for m30, 5/17 patients (29%) had persistent ascites and 7/17 patients (41%) died at 6 months if levels increased, compared to 2/22 patients (9%) and 1/23 patients (4%) with decreasing m30 levels (Table 6). For m65, only 2/24 patients (8%) developed persistent ascites and 3/25 patients (12%) died at 6 months if values decreased, compared to 5/15 patients (33%) for both endpoints if m65 values increased (Table 6). Interestingly, baseline characteristics and the dynamic changes of other prognosis‐relevant parameters such as the MELD score at the early FU visit were similar between patients with a decrease and an increase in epithelial cell death markers. Therefore, increasing epithelial cell death markers at the first evaluation 4–6 weeks of post‐TIPS, but not established prognosis indicators such as the MELD score, were associated with relevant outcome measures like persistent ascites post‐TIPS, mortality or the need for LT within 6 months of post‐TIPS. The stratification of the cohort according to decreasing or increasing values in epithelial cell death markers are displayed in Table S4 for m30 and Table S5 for m65.

**TABLE 6:** A decrease in epithelial cell death markers (Δm30 and Δm65) at early follow‐up a median 35 [27, 28] days after TIPS placement is associated with fewer treatment failures and an improved six‐month transplant‐free survival.

Patients with an early follow‐up: *n* = 40	Persistent ascites (*n* = 7) in patients with an early FU	Rate of six‐month mortality/LT (*n* = 8) in patients with an early FU
Δm30 decrease	2/22, 9.1%	1/23, 4.3%
Δm30 increase	5/17, 29.4%	7/17, 41.2%
Δm65 decrease	2/24, 8.3%	3/25, 12.0%
Δm65 increase	5/15, 33.3%	5/15, 33.3%

*Note:* Data are shown as counts and percentages.

Abbreviations: FU, follow‐up; LT, liver transplantation; TIPS, transjugular intrahepatic portosystemic shunt.

### 3.8 Levels of HMGB‐1 Also Return to Values of Patients With Compensated Cirrhosis

HMGB‐1 is another molecule associated with cellular damage, and therefore, was also evaluated in patients with compensated vs. decompensated liver cirrhosis, and during follow‐up. Unexpectedly, baseline HMGB‐1 values were higher in patients with compensated liver cirrhosis, compared to those with decompensated liver cirrhosis. During follow‐up after TIPS, HMGB‐1 levels gradually increased, ultimately to levels comparable to patients with compensated liver cirrhosis, thus reaching a state of homeostasis. Details on HMGB‐1 values are displayed in Table S6 and Figure S4.

## 4 Discussion

In patients with liver cirrhosis, portal hypertension is a key pathophysiological driver for decompensation and progression to ACLF. The epithelial cell death markers m30 and m65 predict clinical outcomes and disease severity across various stages of liver disease, and thus, may reflect hepatic cellular injury. However, the mechanistic impact of portal hypertension on liver injury and cell death in patients with stable decompensated liver cirrhosis is unknown. Here, we show that portal hypertension is a key driver of cellular, and possibly hepatic injury in patients with ascitic decompensated liver cirrhosis, as its resolution by TIPS induces a decrease of epithelial cell death markers.

Several cross‐sectional studies have shown an association between epithelial cell death markers and liver disease severity. Specifically, m30 and m65 have been established as a diagnostic tool for the severity of steatotic liver disease [11, 17, 29, 30] and primary biliary cholangitis [14]. Epithelial cell death markers have also been evaluated as predictive biomarkers; thus, patients with compensated liver cirrhosis and higher baseline levels of m30/m65 are more likely to develop an acute decompensation (AD) during follow‐up [31]. In patients with decompensated cirrhosis, Macdonald et al. [20] showed a predominance of nonapoptotic cell death with increasing severity of AD and ACLF, most likely of hepatic origin, even in multiorgan failure. Furthermore, higher levels of epithelial cell death markers also predicted the progression from AD to ACLF and the overall 28‐ and 90‐day transplant‐free mortality [20]. Additionally, levels of epithelial cell death markers are also predictive of treatment responses in patients with hepatorenal syndrome (HRS), alcoholic steatohepatitis [32] (ASH) and hepatitis B virus (HBV)‐related ACLF [33].

To date, only a few studies have analysed the reversibility of increased epithelial cell death markers after treatment of the underlying liver disease. Serum levels of epithelial cell death markers have been shown to improve after alcohol withdrawal [19] and HBV treatment [34]. However, the underlying mechanism of ongoing liver injury in patients with decompensated cirrhosis in the absence of a targetable aetiology remains elusive.

Our study is the first to address the question of whether portal hypertension itself induces an increase of liver‐related cell death markers in patients with stable decompensated liver cirrhosis. Decompensated liver cirrhosis is a highly dynamic disease, and ongoing liver injury and the subsequent release of DAMPs are considered to be one of the main perpetuating factors driving further decompensation, ACLF development and finally premature death [35, 36]. In this context, patients receiving a TIPS for ascites present an ideal model to study the effect of portal hypertension on liver outcome‐relevant cellular injury, as these patients undergo TIPS implantation in the absence of an acute decompensating event, such as variceal bleeding or uncontrolled infection. In this study, we confirm that patients with decompensated cirrhosis present with higher baseline values of epithelial cell death markers [20]. Importantly, our data demonstrate that portal hypertension is a key driver of liver‐relevant cellular injury, as its resolution by TIPS attenuates liver outcome‐relevant markers of epithelial cell death. As a proof of principle, we could also show that this beneficial effect only occurs in patients with ascites resolution by TIPS, thus presenting with a clinical response, but not in individuals with TIPS refractory decompensation. Importantly, the effect of portal hypertension on liver‐relevant cellular injury is easily overlooked by traditional serum markers of hepatic injury, as both GPT and GOT are usually not elevated in stable decompensated liver cirrhosis and do not change after TIPS insertion, as also documented in our findings.

Another molecule associated with cellular damage, which was analysed in this study, was HMGB‐1. In general, these results were less clear, as levels of HMGB‐1 were unexpectedly higher in patients with compensated cirrhosis, than patients with decompensated cirrhosis pre‐TIPS. However, during follow‐up, HMGB‐1 levels increased after TIPS, reaching levels comparable to the ones of patients with compensated cirrhosis. Therefore, even though the association of HMGB‐1 to the compensation status is inverse, the dynamic changes are similar to the ones observed for m30/m65. These counterintuitive results may be explained by both pro‐ and anti‐inflammatory properties of HMGB‐1 that are dependent on the redox state [37]. The ELISA kit used in this study does not differentiate between the two redox states, of which one possesses pro‐ and the other anti‐inflammatory capacities. Therefore, it is possible that the anti‐inflammatory redox state is more prevalent in patients with compensated cirrhosis, so that recompensation by TIPS increases rather the anti‐inflammatory isoform of HMGB‐1.

Finally, we also evaluated the capacity of baseline m30/m65 to predict post‐TIPS transplant‐free survival. Novel biomarkers are urgently needed, as about 20%–30% of patients that receive a TIPS for refractory ascites show a clinical nonresponse, which is accompanied by a drastically reduced post‐TIPS survival rate [26, 38, 39]. Previous studies have demonstrated that higher m30/m65 serum levels are independently associated with a poorer overall survival in patients with liver cirrhosis [17, 19, 40] and that keratin 18 cell death markers are also negatively correlated with transplant‐free survival in cirrhosis patients with acute decompensation or acute‐on chronic liver failure [20]. In this context, it is conceivable that the amount of hepatocellular injury before TIPS may play a pathophysiological role due to the profound perfusion changes evoked by TIPS implantation [39]. Similarly, in our cohort, patients with persistent ascites post‐TIPS and patients who did not survive 6 months post‐TIPS presented with higher baseline values of both m30 and m65. However, on multivariable analysis, neither baseline m30 nor m65 values were associated with the cumulative endpoint of liver transplantation or death at 6 months. Surprisingly, we could also not confirm the prognostic association of either, MELD or FIPS score, with transplant‐free survival in our data that had been demonstrated in other cohorts [41]. This may be due to the rather homogeneous composition of our cohort. On the other hand, it also suggests that the baseline level of hepatic cell death does not influence post‐TIPS outcome when pre‐TIPS patient selection adheres to traditional criteria like bilirubin levels < 3 mg/dL [42]. However, even though baseline m30/m65 values did not further predict TIPS response, the dynamic changes of both m30 and m65 post‐TIPS demonstrated robust clinical relevance, as patients with decreasing values post‐TIPS were less likely to develop persistent ascites or die at 6 months, compared to patients with increasing values post‐TIPS. This supports the concept that breaking the postulated vicious circle of portal decompensation and cellular injury by relieving portal pressure translates into an improved clinical outcome. Additionally, the relevance of m30 and m65 fragments may exceed mere diagnostic indication of epithelial injury in this context. In fact, the release of DAMPs in sterile liver injury has been shown to cause immune‐cell recruitment and induction of inflammatory mediators, which is thought to contribute to the hyperinflammatory state seen in decompensated liver cirrhosis, and may therefore serve as a self‐perpetuating mechanism [6, 7, 36, 43]. Consequently, with DAMPs being an integral part of the pathophysiological cascade, their removal is part of a novel liver dialysis device for patients with ACLF that has shown promising preliminary treatment results [44].

In general, our results further support the concept of recompensation that was introduced by the Baveno VII consensus meeting in 2022 [45]. First studies have shown that about 25% of patients achieve recompensation according to the outlined criteria at one year post‐TIPS [28, 46], and recompensated patients have a similar transplant‐free survival compared to patients with compensated (and never decompensated) cirrhosis [46]. Our study provides a pathophysiological explanation for these observations, as we show that portal hypertension itself is a reason for ongoing epithelial, likely hepatic, cell death. In this context, it would also be interesting to further dissect the interplay between epithelial cell death and platelet activation, as recent studies have shown that platelet aggregation is increased [47] and that reticulated platelets are hyperactivated in cirrhosis [48], which also translated into a higher incidence of further decompensation and ACLF development.

Despite its prospective design, our study has several limitations. First, the number of patients is rather low, which especially limits the analysis on the predictive power of baseline m30/m65 values. Furthermore, we present a rather homogeneous cohort so that no conclusion on the additional predictive capacity of epithelial cell death markers in other relevant clinical scenarios can be drawn, for example, TIPS placement for variceal haemorrhage. However, as the main aim of this study was to address the impact of portal hypertension on liver‐relevant cell death in a proof‐of principle design, the rather homogeneous cohort helps to reduce possible other confounders. In addition, it should be noted that m30 and m65 fragments are not exclusively released from the liver, but also from other epithelial tissues, such as the lungs or the intestines. However, overwhelming evidence links m30 and m65 as robust surrogate parameters to clinical, liver injury‐related outcome events, and a likely hepatic origin has been established even in multiorgan failure [20].

In conclusion, our data indicate that, in decompensated liver cirrhosis, correction of portal hypertension by TIPS breaks the vicious cycle of ongoing liver injury, which in turn drives further decompensation, ACLF development and premature death. The resolution of portal hypertension by TIPS implantation appears to facilitate hepatic recompensation by overcoming the unfavourable spontaneous prognosis, otherwise indicated by elevated baseline m30/m65 levels. Our data add to growing evidence that portal hypertension itself, being at the basis of deteriorating events, is a key therapeutic target in the treatment of liver cirrhosis to prevent disease progression.

## Ethics Statement

This study was approved by the local ethics committee (Ärztekammer Hamburg, reference number PV5727).

## Consent

All patients gave written informed consent prior to inclusion.

## Conflicts of Interest

The authors declare no conflicts of interest.

## Supporting information


Data S1.

## Data Availability

Anonymous data are available upon request.
