# Supplementary material for: Recompensation of Liver Cirrhosis by TIPS Reduces Epithelial Cell Death Markers, Translating Into Improved Clinical Outcome
Source: Liver Int. 2024 Nov 12;45(4):e16156. doi: 10.1111/liv.16156 (PMC11897859; doi:10.1111/liv.16156)
Supplement: Supplementary file 1 — Data S1. [file LIV-45-0-s001.docx]

**Recompensation of liver cirrhosis by TIPS reduces epithelial cell death markers, translating into improved clinical outcome**

Felix Piecha^1,2^, Beatrice-Victoria Jahn^1^, Johannes Köntopf^1^, Anja Koop^1^, Ann-Kathrin Ozga^3^, Amirah Al-Jawazneh^1,4^, Aenne Harberts^1^, Christoph Riedel^5^, Peter Buggisch^6^, Daniel Benten^1,7^, Peter Hübener^1^, Gerhard Adam^5^, Samuel Huber^1^, Ansgar W. Lohse^1^, Peter Bannas^5^ and Johannes Kluwe^1^

^1^I. Department of Medicine, University Medical Center Hamburg-Eppendorf, Hamburg, Germany

^2^German Center for Infection Research (DZIF), Partner Site Hamburg-Lübeck-Borstel-Riems, Hamburg, Germany

^3^Center for Experimental Medicine, Institute of Medical Biometry and Epidemiology, University Medical Center Hamburg-Eppendorf, Hamburg, Germany

^4^Protozoa Immunology, Bernhard Nocht Institute for Tropical Medicine, Hamburg, Germany

^5^Department of Diagnostic and Interventional Radiology and Nuclear Medicine, University Medical Center Hamburg-Eppendorf, Hamburg, Germany

^6^ifi-institute for interdisciplinary medicine, Hamburg, Germany

^7^Department of Gastroenterology, Asklepios Hospital Harburg, Hamburg, Germany

**Supplemental Table 1. Baseline characteristics and outcome parameters of the control group of 20 patients with compensated liver cirrhosis**

| **Baseline characteristics** | **Compensated liver cirrhosis, n = 20 (%)** |
| --- | --- |
| Sex: male / female (n, %) | 15 (75.0) / 5 (25.0) |
| Age [y] | 63.0 (54.2, 70.6) |
| Etiology (n, %) |  |
| ALD | 13 (65.0) |
| NASH | 3 (15.0) |
| Cryptogenic | 2 (10.0) |
| Other | 2 (10.0) |
| Ongoing alcohol consumption (n, %) | 2 (10.0) |
| History of SBP (n, %) | 1 (5.0) |
| History of HRS (n, %) | 1 (5.0) |
| History of HE (n, %) | 5 (25.0) |
| History of upper GI bleeding (n, %) | 1 (5.0) |
| Variceal size: 0 / I / II / III / IV / n/a (n, %) | 5 (25.0) / 10 (50.0) / 3 (15.0) / 0 (0.0) / 0 (0.0) / 2 (10.0) |
| Red spots: yes / no / unknown (n, %) | 1 (5.0) / 14 (70.0) / 5 (25.0) |
| Child-Pugh stage A / B / C (n, %) | 18 (90.0) / 2 (10.0) / 0 (0) |
| Child-Pugh score [points] | 5.0 (5.0, 6.0) |
| MELD score [points] | 8.0 (6.3, 10.8) |
| FIPS score [points] | -0.68 (-1.24, -0.12) |
| Liver stiffness [kPa] | 26.0 (16.1, 68.9) |
| CAP [dB/m] | 258.0 (210.8, 327.0) |
| **Outcome parameters** |  |
| Ascites development: yes / no / n/a (n, %) | 0 (0.0) / 19 (95.0) / 1 (5.0) |
| Incidence of HE requiring hospitalization (n, %) | 0 (0.0) |
| Incidence of infections req. hospitalization (n, %) | 0 (0.0) |
| Incidence of HCC (n, %) | 1 (5.0) |
| Incidence of liver transplantations (n, %) | 0 (0.0) |
| Incidence of death (n, %) | 2 (10.0) |
| Transplant-free survival [mo] | 19.5 (11.2, 25.5) |

Table legend: Data are shown as counts and percentages as indicated or median values with the 0.25- and 0.75-quartile.

Abbreviations: ALD, alcoholic liver disease; NASH, non-alcoholic liver disease; SBP, spontaneous bacterial peritonitis; HRS, hepatorenal syndrome; HE, hepatic encephalopathy; GI, gastrointestinal; MELD, model for end-stage liver disease; FIPS, Freiburg Index of post-TIPS survival; CAP, controlled attenuation parameter; HCC, hepatocellular carcinoma.

**Supplemental Table 2. Correlation analysis of baseline parameters and pre-TIPS levels of epithelial cell death markers**

| **Parameter** | **Baseline m30** | **Baseline m65** | **Baseline m30/m65 index** |
| --- | --- | --- | --- |
| Baseline m30 |  | 0.678 | 0.545 |
| Baseline m65 | 0.678 |  | -0.153 |
| Baseline m30/m65 index | 0.545 | -0.153 |  |
| Baseline bilirubin | 0.226 | 0.156 | 0.114 |
| Baseline GOT | 0.309 | 0.347 | 0.009 |
| Baseline GPT | 0.234 | 0.320 | -0.057 |
| Baseline gGT | 0.205 | 0.347 | -0.105 |
| Baseline AP | 0.132 | 0.187 | -0.073 |
| Baseline albumin | -0.086 | -0.084 | 0.051 |
| Baseline platelets | -0.184 | -0.215 | -0.011 |
| Baseline INR | 0.016 | 0.029 | -0.008 |
| Baseline creatinine | 0.248 | 0.277 | -0.022 |
| Baseline CRP | -0.122 | -0.064 | -0.153 |
| Baseline WBCC | -0.127 | -0.089 | -0.095 |
| Baseline CLIF-C AD score | 0.035 | 0.143 | -0.165 |
| Baseline MELD score | 0.222 | 0.244 | 0.008 |
| Baseline FIPS score | 0.249 | 0.287 | -0.084 |
| Baseline Child-Pugh score | 0.266 | 0.150 | 0.195 |
| Portal pressure pre-TIPS | 0.189 | 0.158 | 0.130 |
| Portal pressure post-TIPS | 0.073 | 0.016 | 0.168 |
| PSPG pre-TIPS | 0.181 | 0.141 | 0.080 |
| PSPG post-TIPS | 0.139 | 0.205 | 0.017 |

Table legend: While m30 and m65 showed a relevant correlation with each other, no relevant correlation was found with baseline laboratory values and pressure parameters. Statistical analysis was carried out using Spearman’s Rho.

Abbreviations: INR, international normalized ratio; CRP, C-reactive protein; WBCC, white blood cell count; CLIF-C AD, chronic liver failure consortium acute decompensation, MELD, model for end-stage liver disease; FIPS, Freiburg Index of post-TIPS survival; TIPS, transjugular intrahepatic portosystemic shunt; PSPG, portosystemic pressure gradient.

| **Baseline characteristics** | **Six-month non-survivors (n = 19)** | **Six-month survivors (n = 47)** |
| --- | --- | --- |
| Sex: male / female (n, %) | 14 (73.7) / 5 (26.3) | 22 (46.8) / 25 (53.2) |
| Age [y] | 61.0 (57.0, 69.0) | 57.0 (52.0, 65.0) |
| Etiology – ALD (n, %) | 13 (68.4) | 33 (70.2) |
| Ongoing alcohol consumption (n, %) | 1 (5.3) | 9 (19.1) |
| Time lapse diagnosis cirrhosis – TIPS [mo] | 34.3 (8.4, 79.7) | 12.3 (7.6, 60.7) |
| Time lapse first ascites – TIPS [wk] | 26.8 (16.0, 59.6) | 29.7 (10.6, 51.7) |
| Paracentesis frequency [nr / mo] | 2.0 (1.5, 4.0) | 2.0 (1.0, 2.0) |
| Child-Pugh stage A / B / C (n, %) | 0 (0.0) / 11 (57.9) / 8 (42.1) | 0 (0.0) / 40 (85.1) / 7 (14.9) |
| Child-Pugh score [points] | 9.0 (8.0, 10.0) | 8.0 (8.0, 9.0) |
| MELD score [points] | 14.0 (11.0, 18.0) | 12.0 (9.0, 15.0) |
| CLIF-C AD score [points] | 48.0 (45.0, 55.0) | 49.0 (44.0, 51.0) |
| FIPS score [points] | 0.42 (0.03, 0.79) | -0.15 (-0.81, 0.46) |
| PSPG pre-TIPS [mmHg] | 25.0 (23.0, 29.0) | 25.0 (20.0, 27.5) |
| PSPG post-TIPS [mmHg] | 9.5 (6.0, 13.0) | 10.0 (8.0, 13.0) |
| Δ PSPG: absolute values [mmHg] | 15.5 (13.0, 18.3) | 13.0 (10.0, 20.0) |
| Δ PSPG: percentage | -62.0 (-72.0, -53.2) | -60.0 (-71.4, -46.0) |
| **Epithelial cell death markers** |  |  |
| Baseline m30 [U/l] | 219.0 (184.4, 338.1) | 189.0 (149.4, 261.0) |
| Baseline m65 [U/l]: | 635.8 (479.1, 900.9) | 549.5 (407.8, 883.3) |
| Baseline m30:m65 ratio (apoptotic index) | 0.36 (0.26, 0.55) | 0.36 (0.28, 0.45) |
| **Outcome parameters** |  |  |
| Any paracentesis after TIPS (yes / no / n/a) | 14 (73.7) / 1 (5.2) / 4 (21.1) | 26 (55.3) / 21 (44.7) / 0 (0.0) |
| Refractory ascites (yes / no / n/a) | 12 (63.2) / 3 (15.8) / 4 (21.0) | 1 (2.1) / 45 (95.8) / 1 (2.1) |
| Incidence of infections req. hospitalizaiton | 11 (57.9) | 13 (27.7) |
| Incidence of SBP | 3 (15.8) | 3 (6.4) |
| Incidence of liver transplantations | 3 (15.8) | 0 (0.0) |
| Transplant-free survival [mo. median w/ IQR] | 2.2 (1.1, 5.0) | 19.5 (13.3, 25.7) |

**Supplemental Table 3. Subpopulation analysis of patients stratified according to six-month transplant-free survival**

Table legend: Data are shown as counts and percentages as indicated or median values with the 0.25- and 0.75-quartile. Six-month non-survivors post-TIPS presented with a higher MELD and FIPS score and overall higher values of epithelial cell death markers.

Abbreviations: ALD, alcoholic liver disease; TIPS, transjugular intrahepatic portosystemic shunt; MELD, model for end-stage liver disease; CLIF-C AD, chronic liver failure consortium acute decompensation; FIPS, Freiburg Index of post-TIPS survival; PSPG, portosystemic pressure gradient; SBP, spontaneous bacterial peritonitis.

**Supplemental Table 4. Stratification of the cohort according to dynamic changes in m30 values at the early FU visit.**

| **Baseline characteristics** | **m30 decrease at early FU (n = 23)** | **m30 increase at early FU (n = 17)** |
| --- | --- | --- |
| Sex: male / female (n, %) | 10 (43.5) / 13 (56.5) | 8 (47.1) / 9 (52.9) |
| Age [y] | 55.0 (50.5, 59.5) | 63.0 (57.0, 69.0) |
| Etiology – ALD (n, %) | 15 (65.2) | 12 (70.5) |
| Ongoing alcohol consumption (n, %) | 4 (17.4) | 0 (0.0) |
| Time lapse diagnosis cirrhosis – TIPS [mo] | 12.8 (7.3, 58.6) | 22.6 (9.2, 43.5) |
| Time lapse first ascites – TIPS [wk] | 20.9 (8.8, 38.0) | 39.4 (20.6, 51.7) |
| Paracentesis frequency [nr / mo] | 1.5 (1.0, 2.0) | 2.0 (1.0, 3.0) |
| Child-Pugh stage A / B / C (n, %) | 0 (0.0) / 18 (78.3) / 5 (21.7) | 0 (0.0) / 12 (70.6) / 5 (29.4) |
| Child-Pugh score [points] | 9.0 (8.0, 9.0) | 9.0 (8.0, 10.0) |
| MELD score [points] | 13.0 (10.0, 16.0) | 13.0 (10.0, 17.0) |
| CLIF-C AD score [points] | 49.0 (43.5, 51.0) | 49.0 (46.0, 51.0) |
| FIPS score [points] | -0.2 (-0.6, 0.3) | 0.1 (-0.2, 0.6) |
| PSPG pre-TIPS [mmHg] | 25.0 (22.0, 28.5) | 25.0 (20.0, 27.0) |
| PSPG post-TIPS [mmHg] | 10.5 (9.0, 13.0) | 9.0 (7.0, 13.0) |
| Δ PSPG: absolute values [mmHg] | 14.0 (11.5, 15.8) | 14.0 (10.0, 18.0) |
| Δ PSPG: percentage | -59.1 (-68.3, -48.2) | -60.6 (-70.0, -43.8) |
| **Epithelial cell death markers** |  |  |
| Δ m30 [U/l] | -53.6 (-159.2, -40.4) | 24.6 (9.8, 98.6) |
| Δ m30 % [U/l] | -25.4 (-43.1, -20.0) | 12.7 (6.1, 49.1) |
| **Laboratory values** |  | |
| Baseline Bilirubin [mg/dl] | 1.6 (1.1, 1.9) | 1.1 (0.5, 2.2) |
| Bilirubin at early FU [mg/dl] | 1.8 (1.4, 2.6) | 1.3 (0.7, 2.2) |
| Baseline creatinine [mg/dl] | 0.9 (0.8, 1.3) | 1.2 (0.7, 1.4) |
| Creatinine at early FU [mg/dl] | 0.9 (0.7, 1.0) | 1.1 (0.7, 1.3) |
| Baseline albumin [g/l] | 26.6 (23.2, 29.4) | 26.4 (24.8, 31.9) |
| Albumin at early FU [g/l] | 25.4 (22.7, 28.8) | 25.5 (19.5, 29.4) |
| Baseline C-reactive protein [mg/l] | 11.0 (5.0, 22.5) | 19.0 (7.0, 28.0) |
| C-reactive protein at early FU [mg/l] | 5.0 (4.8, 14.0) | 12.0 (5.5, 32.5) |
| Baseline MELD score [points] | 13.0 (10.0, 16.0) | 13.0 (10.0, 17.0) |
| MELD score at early FU [points] | 14.0 (11.5, 15.0) | 12.0 (9.0, 15.0) |
| **Outcome parameters** |  |  |
| Any paracentesis after TIPS (yes / no / n/a) | 15 (65.2) / 8 (34.8) / 0 (0.0) | 12 (70.6) / 5 (29.4) / 0 (0.0) |
| Refractory ascites (yes / no / n/a) | 2 (8.7) / 20 (87.0) / 1 (4.3) | 5 (29.4) / 12 (70.6) / 0 (0.0) |
| Incidence of infections req. hospitalizaiton | 9 (39.1) | 5 (29.4) |
| Incidence of SBP | 2 (8.7) | 1 (5.9) |
| Incidence of LT at six months | 0 (0.0) | 2 (11.8) |
| Incidence of death at six months | 1 (4.3) | 5 (29.4) |
| Transplant-free survival [mo] | 18.4 (12.9, 26.5) | 11.2 (5.1, 25.8) |

Table legend: Data are shown as counts and percentages as indicated or median values with the 0.25- and 0.75-quartile. Except m30 dynamics, baseline characteristics, baseline values and dynamic changes of other prognosis-relevant parameters were similar between the cohorts.

Abbreviations: FU, follow-up; ALD, alcoholic liver disease; TIPS, transjugular intrahepatic portosystemic shunt; MELD, model for end-stage liver disease; CLIF-C AD, chronic liver failure consortium acute decompensation; FIPS, Freiburg Index of post-TIPS survival; PSPG, portosystemic pressure gradient; SBP, spontaneous bacterial peritonitis; LT, liver transplantation.

**Supplemental Table 5. Stratification of the cohort according to dynamic changes in m65 values at the early FU visit.**

| **Baseline characteristics** | **m65 decrease at early FU (n = 25)** | **m65 increase at early FU (n = 15)** |
| --- | --- | --- |
| Sex: male / female (n, %) | 12 (48.0) / 13 (52.0) | 6 (40.0) / 9 (60.0) |
| Age [y] | 55.0 (50.0, 65.0) | 63.0 (57.0, 65.5) |
| Etiology – ALD (n, %) | 15 (60.0) | 11 (73.3) |
| Ongoing alcohol consumption (n, %) | 4 (16.0) | 0 (0.0) |
| Time lapse diagnosis cirrhosis – TIPS [mo] | 16.3 (7.6, 53.1) | 19.1 (8.7, 71.6) |
| Time lapse first ascites – TIPS [wk] | 27.9 (9.7, 44.6) | 34.0 (20.2, 52.2) |
| Paracentesis frequency [nr / mo] | 1.5 (1.0, 2.0) | 2.0 (1.0, 3.5) |
| Child-Pugh stage A / B / C (n, %) | 0 (0.0) / 20 (80.0) / 5 (20.0) | 0 (0.0) / 10 (66.7) / 5 (33.3) |
| Child-Pugh score [points] | 9.0 (8.0, 9.0) | 9.0 (8.0, 10.0) |
| MELD score [points] | 13.0 (10.0, 16.0) | 13.0 (10.5, 20.0) |
| CLIF-C AD score [points] | 49.0 (43.0, 51.0) | 49.0 (46.0, 53.5) |
| FIPS score [points] | -0.2 (-0.6, 0.4) | 0.1 (-0.7, 0.8) |
| PSPG pre-TIPS [mmHg] | 24.0 (22.0, 27.0) | 25.0 (20.0, 28.5) |
| PSPG post-TIPS [mmHg] | 10.5 (8.5, 13.0) | 9.0 (7.5, 12.5) |
| Δ PSPG: absolute values [mmHg] | 14.0 (10.8, 16.0) | 14.0 (10.5, 19.0) |
| Δ PSPG: percentage | -59.1 (-70.6, -47.6) | -60.6 (-69.2, -45.2) |
| **Epithelial cell death markers** |  |  |
| Δ m65 [U/l] | -122.9 (-313.6, -74.7) | 85.3 (57.3, 190.5) |
| Δ m65 % [U/l] | -22.2 (-39.1, -14.4) | 19.3 (12.9, 32.8) |
| **Laboratory values** |  | |
| Baseline Bilirubin [mg/dl] | 1.6 (1.0, 1.9) | 1.1 (0.5, 2.3) |
| Bilirubin at early FU [mg/dl] | 1.8 (1.3, 2.5) | 1.3 (0.7, 2.2) |
| Baseline creatinine [mg/dl] | 0.9 (0.8, 1.2) | 1.2 (0.7, 1.5) |
| Creatinine at early FU [mg/dl] | 0.9 (0.8, 1.0) | 1.2 (0.7, 1.6) |
| Baseline albumin [g/l] | 26.3 (23.1, 29.1) | 26.8 (25.0, 32.6) |
| Albumin at early FU [g/l] | 24.4 (21.7, 28.7) | 25.6 (22.0, 30.3) |
| Baseline C-reactive protein [mg/l] | 11.0 (5.0, 25.0) | 19.0 (8.5, 31.5) |
| C-reactive protein at early FU [mg/l] | 5.0 (4.3, 13.8) | 14.0 (9.0, 24.0) |
| Baseline MELD score [points] | 13.0 (10.0, 16.0) | 13.0 (10.5, 20.0) |
| MELD score at early FU [points] | 13.0 (11.0, 15.0) | 12.0 (10.0, 19.0) |
| **Outcome parameters** |  |  |
| Any paracentesis after TIPS (yes / no / n/a) | 15 (60.0) / 10 (40.0) / 0 (0.0) | 12 (80.0) / 3 (20.0) / 0 (0.0) |
| Refractory ascites (yes / no / n/a) | 2 (8.0) / 22 (88.0) / 1 (4.0) | 5 (33.3) / 10 (66.7) / 0 (0.0) |
| Incidence of infections req. hospitalization | 9 (36.0) | 5 (33.3) |
| Incidence of SBP | 2 (8.0) | 1 (6.7) |
| Incidence of LT at six months | 1 (4.0) | 1 (6.7) |
| Incidence of death at six months | 2 (8.0) | 4 (26.7) |
| Transplant-free survival [mo] | 18.4 (12.0, 27.5) | 11.9 (5.0, 24.4) |

Table legend: Data are shown as counts and percentages as indicated or median values with the 0.25- and 0.75-quartile. Except m65 dynamics, baseline characteristics, baseline values and dynamic changes of other prognosis-relevant parameters were similar between the cohorts.

Abbreviations: FU, follow-up; ALD, alcoholic liver disease; TIPS, transjugular intrahepatic portosystemic shunt; MELD, model for end-stage liver disease; CLIF-C AD, chronic liver failure consortium acute decompensation; FIPS, Freiburg Index of post-TIPS survival; PSPG, portosystemic pressure gradient; SBP, spontaneous bacterial peritonitis; LT, liver transplantation.

**Supplemental Table 6. HMGB-1 values in patients with compensated liver cirrhosis (n=20), and decompensated liver cirrhosis (pre-TIPS, n=53) and during follow-up**

| **Baseline levels of HMGB-1 and changes during follow-up** | | | | |
| --- | --- | --- | --- | --- |
|  | **Compensated**  (n = 20) | **Decompensated**  Pre-TIPS (n = 53) | **Early follow-up**  (n = 39) | **Late follow-up**  (n = 32) |
| HMGB-1 [U/l] | 27.2 (7.4, 38.5) | 12.9 (6.5, 26.5) | 20.8 (10.0, 43.0) | 23.9 (9.4, 51.2) |
| ΔHMGB-1 [U/l] |  |  | 6.9 (-4.5, 31.5) | 5.9 (-6.8, 31.8) |
| ΔHMGB-1 % [U/l] |  |  | 79.3 (-25.7, 207.1) | 58.9 (-18.9, 285.1) |
| **Stratification according to ascites control post-TIPS** | | | | |
|  | Persistent ascites (n = 10) | | Ascites control (n = 40) | |
| Baseline HMBG-1 [U/l] | 13.3 (9.3, 30.8) | | 12.3 (6.1, 23.4) | |
| **Stratification according to six-month survival post-TIPS** | | | | |
|  | Non-survivors (n = 13) | | Survivors (n = 40) | |
| Baseline HMBG-1 [U/l] | 11.3 (6.2, 24.3) | | 13.0 (6.6, 27.1) | |

Table legend: Data are shown as median values with the 0.25- and 0.75-quartile. HMGB-1 levels were relevantly higher in patients with compensated liver cirrhosis compared to patients with decompensated liver cirrhosis. After TIPS, levels of HMGB-1 gradually increased to values comparable of patients with compensated cirrhosis.

Abbreviations: TIPS, transjugular intrahepatic portosystemic shunt; HMGB-1, high-mobility-group-box protein 1.

**Supplemental Fig. 1 Baseline values of epithelial cell death markers stratified according to persistent ascites (n=13) vs. ascites control (n=48) post-TIPS**

Figure legend: Baseline values for both m30 (panel A) and m65 (panel B) tend to be higher in patients with persistent ascites compared to patients with ascites control post-TIPS.

Abbreviations: TIPS, transjugular intrahepatic portosystemic shunt.

**Supplemental Fig. 2 Baseline values of epithelial cell death markers stratified according to six-month transplant-free survival**

Figure legend: Values for both m30 (panel A) and m65 (panel B) tend to be higher in six-month non-survivors (n=19) compared to six-month survivors (n=47).

Abbreviations: TIPS, transjugular intrahepatic portosystemic shunt.


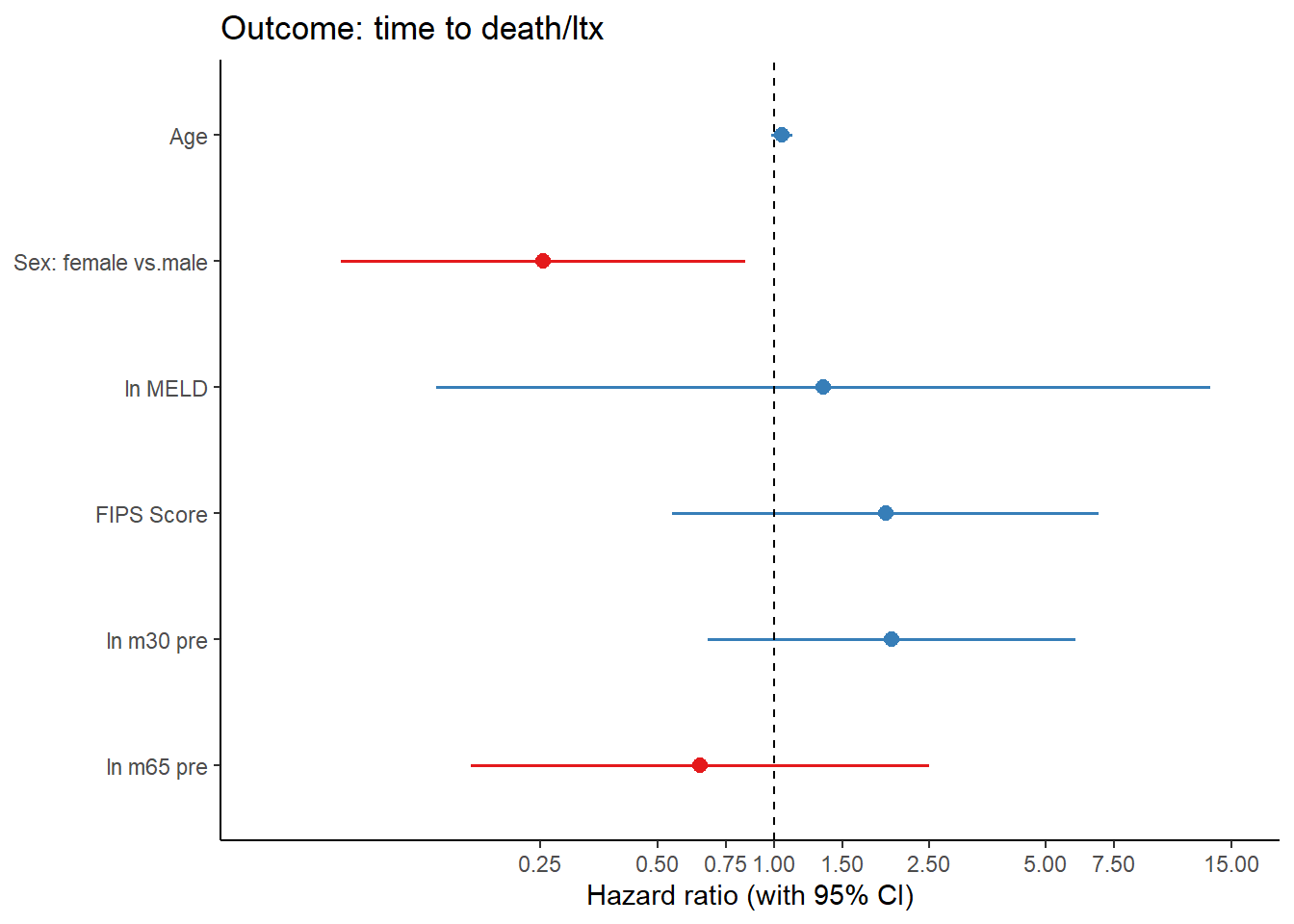


**Supplemental Fig. 3 Hazard ratio for the cumulative endpoint death and liver transplantation at six months**

Figure legend: Except female sex, none of the variables that entered the model had a relevant influence on six-month transplant-free survival.

Abbreviations: MELD, model for end-stage liver disease; FIPS, Freiburg Index of post-TIPS survival.

**Supplemental Fig. 4 HMBG-1 values in patients with compensated and decompensated liver cirrhosis (pre-TIPS) and changes during follow-up**

Figure legend: HMGB-1 levels were higher in patients with compensated liver cirrhosis compared to patients with decompensated liver cirrhosis at baseline After TIPS, levels of HMGB-1 gradually increased to values comparable of patients with compensated cirrhosis.

Abbreviations: TIPS, transjugular intrahepatic portosystemic shunt; HMGB-1, high-mobility-group-box protein 1; FU, follow-up.
